# Selection of pancreaticojejunostomy technique after pancreaticoduodenectomy: duct-to-mucosa anastomosis is not better than invagination anastomosis: A meta-analysis

**DOI:** 10.1097/MD.0000000000012621

**Published:** 2018-10-05

**Authors:** Yunxiao Lyu, Ting Li, Bin Wang, Yunxiao Cheng, Sicong Zhao

**Affiliations:** a Department of Hepatobiliary Surgery; b Department of Personnel Office, Dongyang People's Hospital, Dongyang, Zhejiang Province, China.

**Keywords:** duct-to-mucosa, invagination, meta-analysis, pancreatoduodenectomy, systematic review

## Abstract

**Background:**

One of the most clinically significant current discussions is the optimal pancreaticojejunostomy (PJ) technique for pancreaticoduodenectomy (PD). We performed a meta-analysis to compare duct-to-mucosa and invagination techniques for pancreatic anastomosis after PD.

**Methods:**

A systematic search of PubMed, Embase, Web of Science, the Cochrane Central Library, and ClinicalTrials.gov up to June 1, 2018 was performed. Randomized controlled trials (RCTs) comparing duct-to-mucosa versus invagination PJ were included. Statistical analysis was performed using RevMan 5.3 software.

**Results:**

Eight RCTs involving 1099 patients were included in the meta-analysis. The rate of postoperative pancreatic fistula (POPF) was not significantly different between the duct-to-mucosa PJ (110/547, 20.10%) and invagination PJ (98/552, 17.75%) groups in all 8 studies (risk ratio, 1.13; 95% CI, 0.89–1.44; *P* = .31). The subgroup analysis using the International Study Group on Pancreatic Fistula criteria showed no significant difference in POPF between duct-to-mucosa PJ (97/372, 26.08%) and invagination PJ (78/377, 20.68%). No significant difference in clinically relevant POPF (CR-POPF) was found between the 2 groups (55/372 vs 40/377, *P* = .38). Additionally, no significant differences in delayed gastric emptying, post-pancreatectomy hemorrhage, reoperation, operation time, or length of stay were found between the 2 groups. The overall morbidity and mortality rates were not significantly different between the 2 groups.

**Conclusion:**

The duct-to-mucosa technique seems no better than the invagination technique for pancreatic anastomosis after PD in terms of POPF, CR-POPF, and other main complications. Further studies on this topic are therefore recommended.

## 1 Introduction

Pancreaticoduodenectomy (PD) is a complex, high-risk standard surgical procedure that is indicated primarily for periampullary diseases. Central to the entire discipline of PD are postoperative mortality and morbidity. Although operative mortality in patients undergoing PD has decreased, the incidence of postoperative morbidity remains high at 40% to 50%.^[1–6]^ Postoperative pancreatic fistula (POPF) is the most common complication, with rates ranging from 5% to 30% in previous studies.^[7,8]^ Many methods have been described to decrease the risk of POPF, including the use of medications (prophylactic octreotide,^[9,10]^ sealants^[11]^), prophylactic pancreatic stenting,^[12]^ and improvements in pancreatic reconstruction techniques.^[1,2]^ The most commonly used pancreatic reconstruction techniques are pancreaticogastrostomy (PG) and pancreaticojejunostomy (PJ). Several methods of PJ currently exist, the 2 most common of which are duct-to-mucosa PJ and invagination PJ. In the past few decades, many studies have assessed the safety and efficacy of these 2 methods.^[13–15]^ A major advantage of duct-to-mucosa PJ is that it allows drainage of the main duct into the intestine. Many previous studies have shown a lower incidence of pancreatic fistula after duct-to-mucosa PJ than invagination PJ.^[16–19]^ Therefore, duct-to-mucosa PJ is one of the most widely used PJ methods. Theoretically, however, duct-to-mucosa PJ cannot provide drainage of minor ducts and may require higher-level technology. A previous study demonstrated that invagination PJ could reduce the rate of POPF.^[20]^ However, a recent randomized controlled trial (RCT) showed that invagination PJ was not associated with a lower rate of POPF but was instead associated with a decreased severity of POPF.^[15]^ One of the most clinically significant current discussions is the optimal PJ technique for PD. Increasingly more RCTs have been performed or are ongoing. The aim of this study was to compare the clinical outcomes of duct-to-mucosa PJ and invagination PJ.

## 2 Materials and methods

### 2.1 Search strategy

Two researchers (TL and YXL) independently conducted a comprehensive and systematic search of PubMed, Embase, Web of Science, the Cochrane Central Library, and ClinicalTrials.gov up to June 2018. English search terms included but were not limited to the following: pancreatoduodenectomy, PD, PJ, duct-to-mucosa, and invagination. The search was limited initially to publications of RCTs. The references of the articles identified after the initial search were also manually reviewed. This meta-analysis adhered to the Preferred Reporting Items for Systematic Reviews and Meta-Analyses (PRISMA) statement.

### 2.2 Inclusion and exclusion criteria

The following inclusion criteria were applied: the RCT must have compared the clinical outcomes between duct-to-mucosa PJ and invagination PJ after PD. The participants must have had a clinical diagnosis of POPF. The study must have provided adequate data on the clinical outcomes.

We excluded studies that were non-RCTs, retrospective studies, review articles, case reports, abstract, editorials, and letters to the editor; were repeatedly published by the same author or agency; and had insufficient data on outcome measures.

### 2.3 Clinical outcomes of interest

The primary outcomes were the incidence of POPF and clinically relevant POPF (CR-POPF) after PD. The other outcomes were delayed gastric emptying (DGE), post-pancreatectomy hemorrhage (PPH), reoperation, morbidity, mortality, operation time, and length of stay (LOS).

### 2.4 Data extraction

Two reviewers (YXC and BW) independently extracted the following original data from the literature and entered it onto a standardized form: first author, year of publication, study period, and country where the study took place; sample size, types of PJ, texture and diameter of the pancreas, and definition of POPF. If necessary, the author or authors of the study were contacted to obtain the necessary data. Conflicts in data abstraction were resolved by consensus and by referring to the original article.

### 2.5 Quality assessment

The authors independently assessed the quality of the literature in accordance with the Cochrane Collaboration Handbook.^[21]^ The scoring system included the following criteria: random sequence generation, allocation concealment, blinding of participants and personnel, blinding of the results assessment, incomplete data of the results, selective reporting, and other sources of bias.

### 2.6 Statistical analysis

All included data were assessed using Review Manager (RevMan) version 5.3 software (Cochrane Informatics and Knowledge Management Department, Copenhagen, Kongeriget Danmark). The risk ratio (RR) and 95% confidence interval (CI) were used for dichotomous outcomes. Publication bias was evaluated by the chi-squared test and funnel plots. Heterogeneity among studies was evaluated by the chi-squared test. A two-tailed *P* value of <.05 was considered statistically significant.

### 2.7 Ethics statement

This study was a secondary analysis regarding human subject data published in the public domain; thus, no ethical approval was required.

## 3 Results

### 3.1 Selected studies and characteristics of the trials

**Figure 1:**
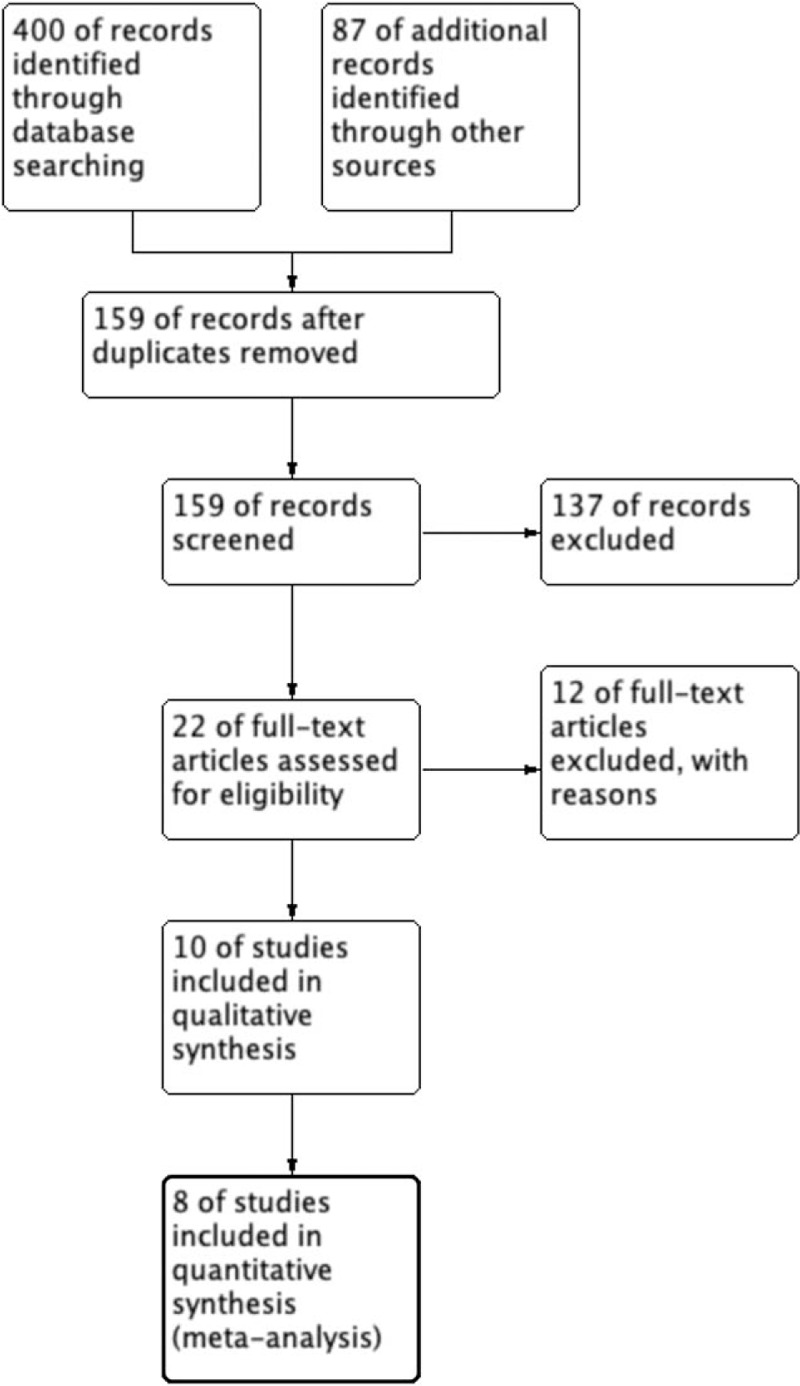
Flow diagram of the published articles evaluated for inclusion in this meta-analysis.

Based on our search criteria, we yielded a total of 487 papers from the respective search engines, of which 320 duplicate articles were excluded. The remaining 159 studies were retrieved for assessment of their titles and abstracts, leaving 8 articles that met the inclusion criteria. Finally, 8 RCTs involving 1099 participants were included in the meta-analysis.^[13–15,20,22–25]^ A detailed flowchart of the selection process is depicted in Fig. 1.

**Figure 2:**
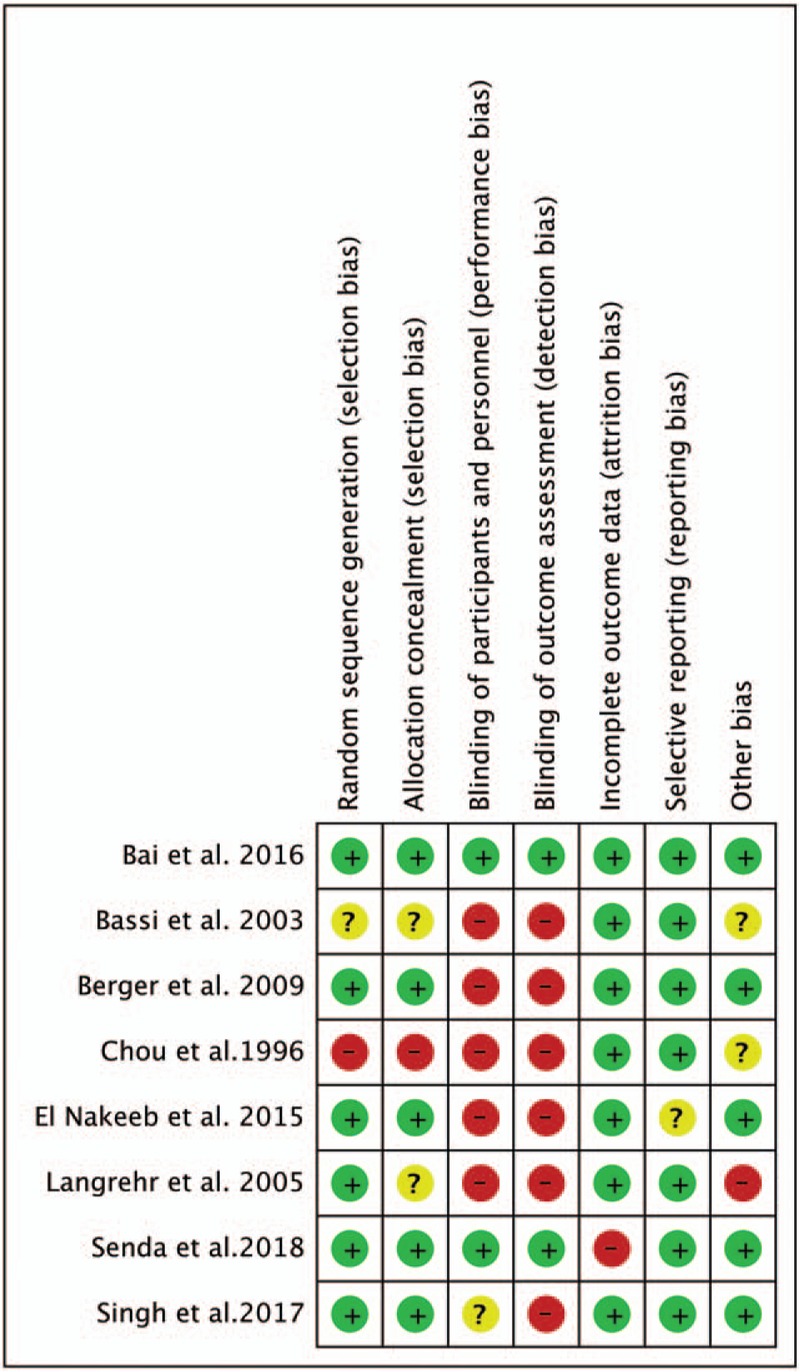
Consensus risk-of-bias assessment of the included studies. Green, low risk; yellow, unclear; red, high risk.

The 1099 patients were divided into the duct-to-mucosa PJ group (n = 547) and invagination group (n = 552). The sample sizes ranged from 92 to 197, and the incidence rate of POPF varied from 3.5% to 32.0%. Of these studies, 5 trials^[15,20,22,24,25]^ provided POPF data using the definition established by the International Study Group on Pancreatic Fistula (ISGPF), and 3 studies^[13,14,23]^ used different definitions of POPF. Data regarding the pancreatic texture were provided in 6 studies,^[13,15,22,24,25]^ and the diameter of the pancreatic duct was provided in 5 studies.^[15,20,22,24,25]^Table 1 shows the main characteristics of the studies included in this meta-analysis, and Table 2 shows the definitions of POPF used in the studies. Figure 2 presents an consensus risk-of bias assessment of the included studies.

**Table 1:**
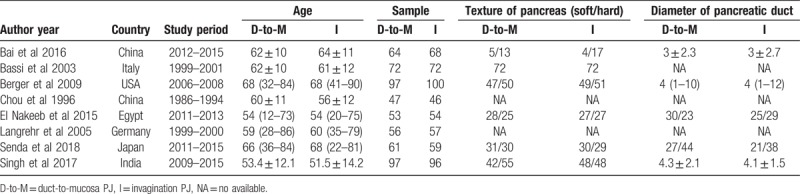
Characteristics of the included studies.

**Table 2:**
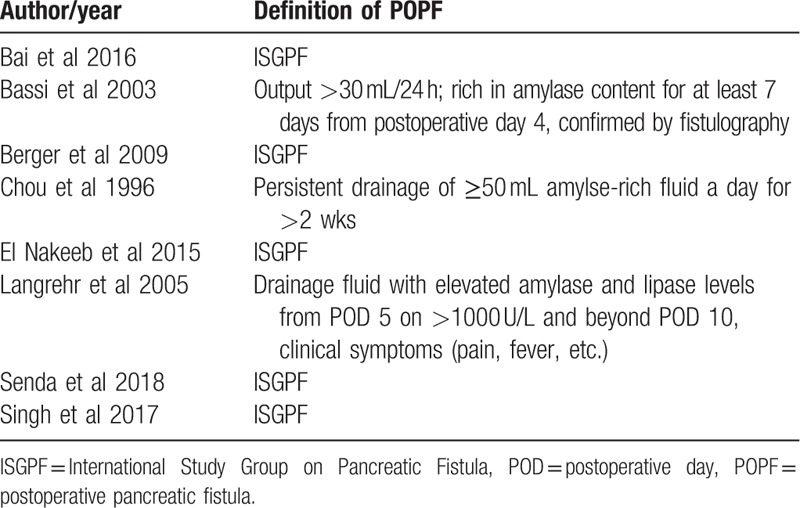
Definition of POPF.

### 3.2 POPF

All 8 trials involving 1099 participants were pooled to compare the incidence of POPF after PD. There were no significant differences between the duct-to-mucosa PJ group (20.1%) and invagination PJ group (17.75%) (RR, 1.13; 95% CI, 0.89–1.44; *P* = .31) (Fig. 3  A). Five studies involving 661 samples using the ISGPF definition showed that there were no significant differences between the duct-to-mucosa group and invagination group (RR, 1.26; 95% CI, 0.97–1.63; *P* = .08) (Fig. 3  A).

**Figure 3:**
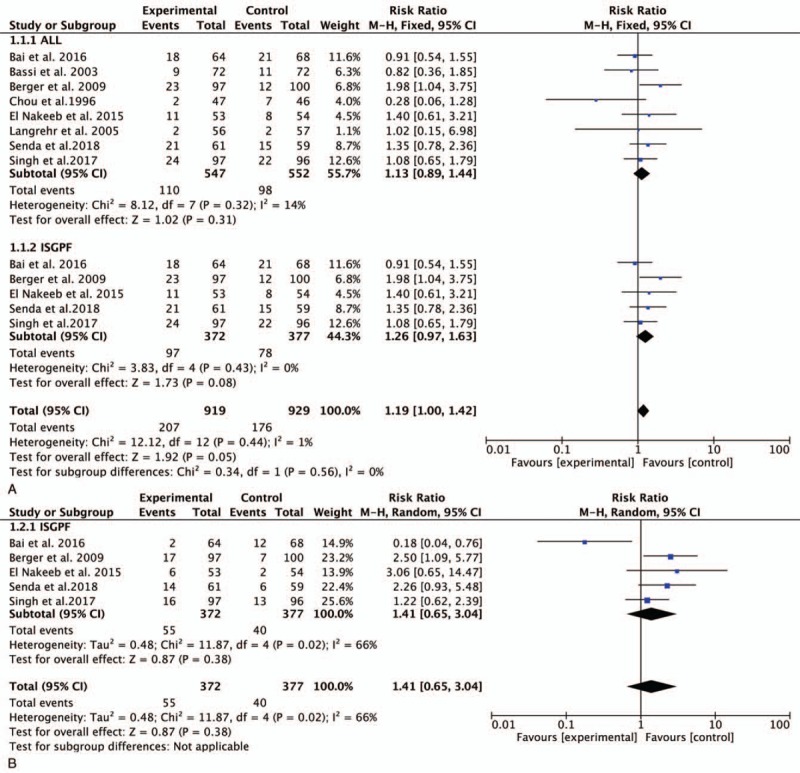
Forest plot of the meta-analysis comparing duct-to-mucosa PJ and invagination PJ with respect to (A) POPF, (B) CR-POPF, (C) DGE, (D) PPH, and (E) reoperation. CR-POPF = clinically relevant POPF, DGE = delayed gastric emptying, PJ = pancreaticojejunostomy, POPF = postoperative pancreatic fistula, PPH = post-pancreatectomy hemorrhage.

**Figure 3 (Continued):**
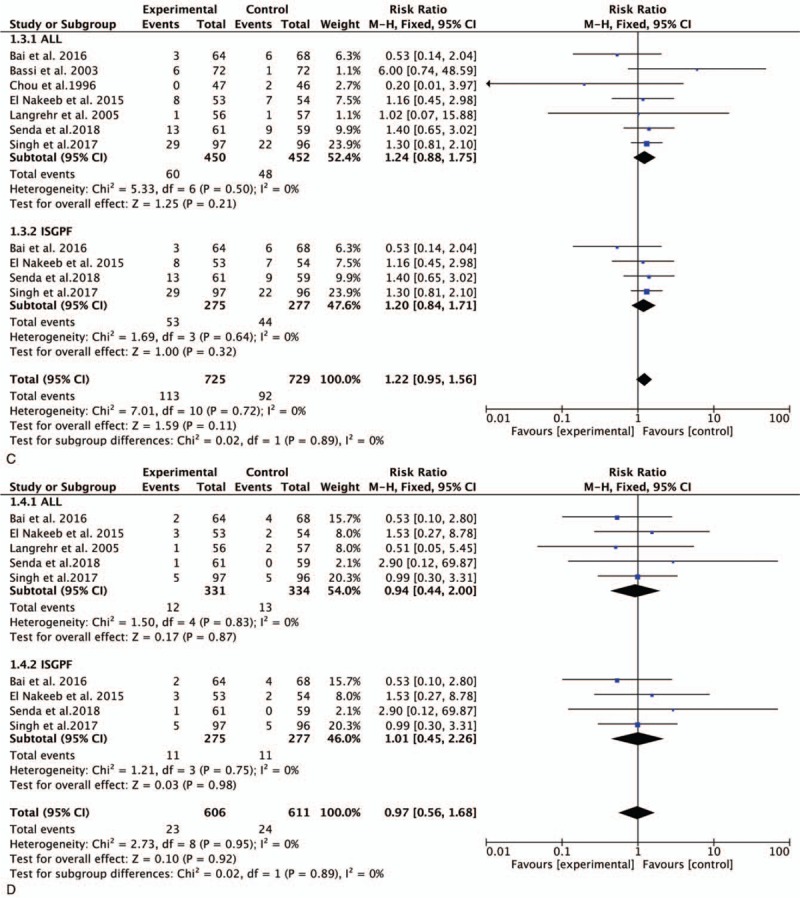
Forest plot of the meta-analysis comparing duct-to-mucosa PJ and invagination PJ with respect to (A) POPF, (B) CR-POPF, (C) DGE, (D) PPH, and (E) reoperation. CR-POPF = clinically relevant POPF, DGE = delayed gastric emptying, PJ = pancreaticojejunostomy, POPF = postoperative pancreatic fistula, PPH = post-pancreatectomy hemorrhage.

**Figure 3 (Continued):**
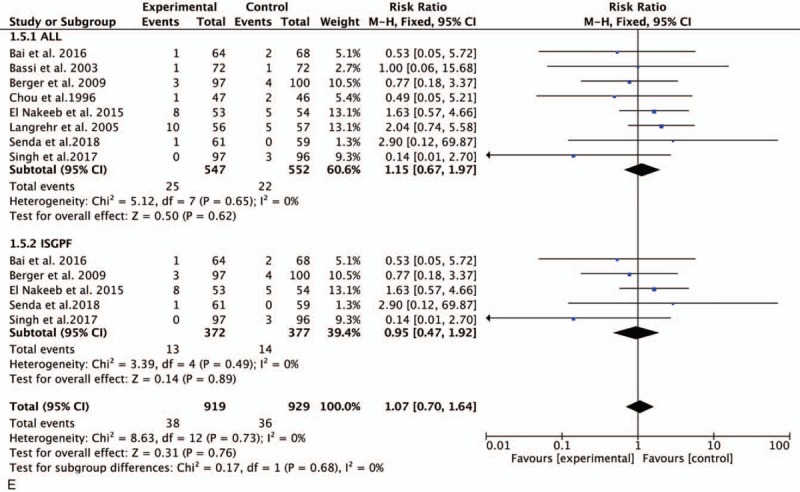
Forest plot of the meta-analysis comparing duct-to-mucosa PJ and invagination PJ with respect to (A) POPF, (B) CR-POPF, (C) DGE, (D) PPH, and (E) reoperation. CR-POPF = clinically relevant POPF, DGE = delayed gastric emptying, PJ = pancreaticojejunostomy, POPF = postoperative pancreatic fistula, PPH = post-pancreatectomy hemorrhage.

### 3.3 CR-POPF

Five trials provided data regarding CR-POPF in accordance with the ISGPF criteria. The pooled data demonstrated no statistically significant difference between the duct-to-mucosa PJ and invagination PJ groups (RR, 1.14; 95% CI, 0.65–3.04; *P* = .38) (Fig. 3  B).

### 3.4 DGE

We calculated the pooled estimates using a random-effects model (*I*^2^ = 0%). Data regarding DGE were provided in 7 studies with 60 of 450 patients in the duct-to-mucosa PJ group and 48 of 452 in the invagination PJ group. There was no significant difference between the 2 groups (RR, 1.24; 95% CI, 0.88–1.76; *P* = .22) (Fig. 3  C). In accordance with the ISGPF criteria, no significant difference was shown in the meta-analysis (RR, 1.22; 95% CI, 0.85–1.75; *P* = .27) (Fig. 3  C).

### 3.5 PPH

In a comparison of the incidence of PPH, we found that 5 studies reported the clinical outcome of interest. The incidence of PPH in the duct-to-mucosa and invagination PJ groups is presented in Fig. 3  D. This meta-analysis demonstrated no significant difference between the 2 PJ techniques (RR, 0.94; 95% CI, 0.44–2.00; *P* = .87) (Fig. 3  D). After stratifying the patients according to the definition of PPH, duct-to-mucosa PJ was not superior to invagination PJ (RR, 1.01; 95% CI, 0.45–2.26; *P* = .98) (Fig. 3  D).

### 3.6 Reoperation

All 8 studies reported the rates of reoperation. No significant difference was found between the 2 groups (RR, 1.15; 95% CI, 0.67–0.1.97; *P* < .62) (Fig. 3  E). Similar results were obtained in the ISGPF analysis (RR, 0.95; 95% CI, 0.47–1.92; *P* = .89) (Fig. 3  E).

### 3.7 Operation time

Data on the operation time were reported in all trials. However, there was no significant difference between the 2 groups in the meta-analysis (mean difference [MD], 22.45; 95% CI,– 7.14–52.04; *P* = .14). The meta-analysis of studies performed in accordance with the ISGPF criteria demonstrated no significant difference between the 2 groups (MD, 26.30; 95% CI, –19.55–72.15; *P* = .26) (Fig. 4 A).

**Figure 4:**
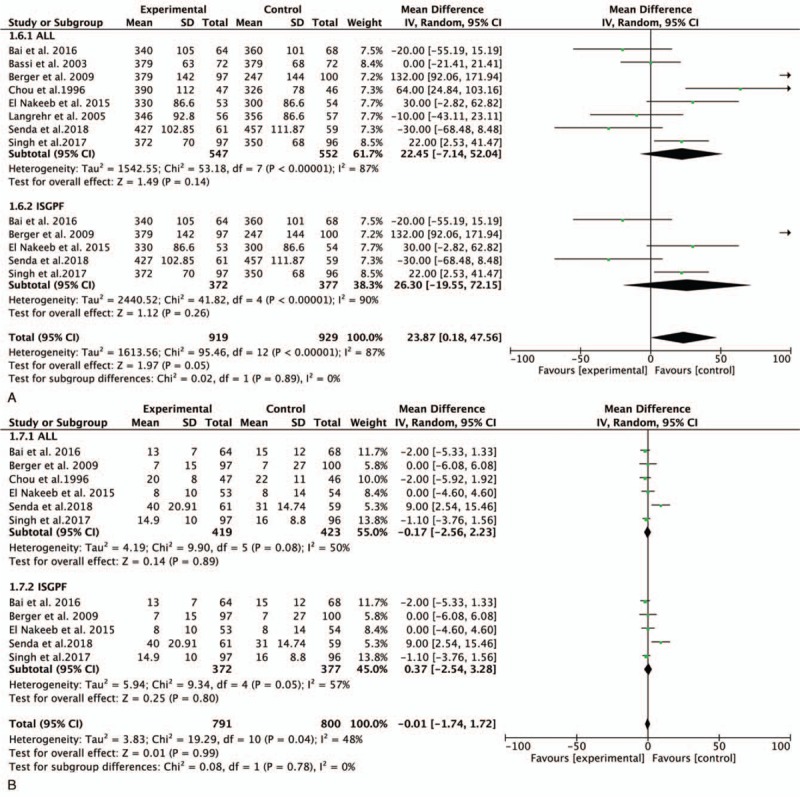
Forest plot of the meta-analysis comparing duct-to-mucosa PJ and invagination PJ with respect to the (A) operation time, (B) LOS, (C) morbidity, and (D) mortality. LOS = length of stay, PJ = pancreaticojejunostomy.

**Figure 4 (Continued):**
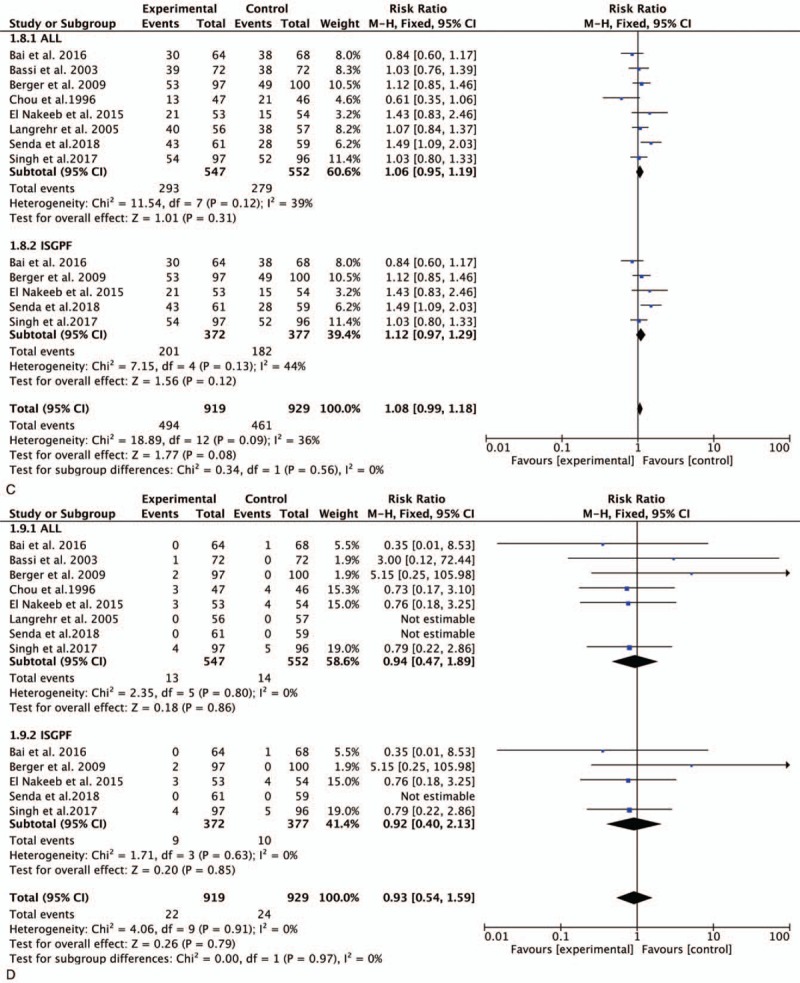
Forest plot of the meta-analysis comparing duct-to-mucosa PJ and invagination PJ with respect to the (A) operation time, (B) LOS, (C) morbidity, and (D) mortality. LOS = length of stay, PJ = pancreaticojejunostomy.

### 3.8 LOS

Six studies involving 842 patients (419 in the duct-to-mucosa PJ group and 423 in the invagination PJ group) were pooled to compare the LOS. There was no significant difference between the 2 groups (MD, –0.17; 95% CI, –2.56–2.23; *P* = .89). Among studies using the ISGPF definition, no significant difference was found between the 2 groups (MD, 0.37; 95% CI, –2.54–3.28; *P* = .80) (Fig. 4 B).

### 3.9 Morbidity

All trials provided data regarding morbidity. The meta-analysis demonstrated no significant difference between the 2 groups (RR, 1.06; 95% CI, 0.95–1.19; *P* = .31) (Fig. 4 D). Analysis of morbidity according to the ISGPF criteria revealed no significant difference between the 2 techniques (RR, 1.12; 95% CI, 0.97–1.29; *P* = .12) (Fig. 4 D).

### 3.10 Mortality

The analysis of mortality was performed using a random-effects model (*I*^2^ = 0%). All studies provided data on mortality rates among 1099 patients. The meta-analysis demonstrated no significant difference between the 2 groups (RR, 0.94; 95% CI, 0.47–1.89; *P* = .86) (Fig. 4 E). Analysis according to the ISGPF criteria did not change the result (RR, 0.92; 95% CI, 0.40–2.13; *P* = .85) (Fig. 4 E).

## 4 Discussion

The optimal reconstruction technique for PJ after PD remains controversial. In the present study, duct-to-mucosa PJ did not seem to be superior to invagination PJ in terms of POPF and CR-POPF. No significant differences in DGE, PPH, or the main clinical outcomes were found between the 2 groups.

The most effective pancreatic construction technique has been debated in many studies.^[2,26,27]^ Two major techniques performed universally are PG and PJ. Although many studies have compared PG with PJ, the best way to reconstruct the pancreas has not been determined.^[28–30]^ PJ is the most commonly used method to restore the pancreatic anastomosis, and its main advantage is that it is more physiological. The surgical techniques of PJ are duct-to-mucosa, invagination, and binding PJ. Binding PJ was proposed in 2002 by Peng et al.^[31]^ Some studies have proposed that binding PJ may reduce the incidence of POPF.^[27,32]^ In the European population, however, binding PJ did not reduce the incidence of POPF.^[33]^ Few clinical studies have been performed to evaluate binding PJ, and the technique may still need some modifications. Therefore, assessment of this technique is not within the scope of the present study.

As mentioned above, the 2 most widely used PJ methods are duct-to-mucosa PJ and invagination PJ. A primary concern of PD is POPF. POPF can lead to intra-abdominal abscess formation, DGE, PPH, and increased morbidity. The occurrence of POPF is multifactorial, and studies have shown that it may be related to obesity, the pancreatic texture, the pancreatic duct diameter, and pancreas reconstruction.^[34–38]^ Previously published studies of the effect of pancreatic reconstruction on POPF are not consistent. The main advantage of duct-to-mucosa PJ is that it assures drainage of the main duct into the intestine. Previous studies involving animals and humans suggest that duct-to-mucosa PJ is associated with a lower incidence of POPF than is invagination PJ.^[17–19,39]^ In the present meta-analysis, 7 studies demonstrated that invagination PJ was associated with a lower incidence of POPF. However, minor ducts and a soft pancreas make duct-to-mucosa PJ difficult. In contrast to duct-to-mucosa PJ, invagination PJ allows for easier reconstruction and has advantages in patients with a soft pancreas. Some studies, including an RCT, demonstrated that invagination PJ was associated with a lower incidence of POPF. Nevertheless, the conclusions of previous relevant research regarding the effects of duct-to-mucosa PJ and invagination PJ on the development of POPF remain controversial.^[40–42]^ A major strength of the present study is the inclusion of 2 recent RCTs. Nonetheless, the results of previous studies are conflicted.

In previous studies, a major source of heterogeneity was the difference in the definition of pancreatic fistula. Before 2005, the definition of POPF was variable among individual studies. The ISGPF organized experts from well-known European, Japanese, Australian, North American, and South American centers in 2005 to establish the definition and classification system of pancreatic fistula.^[43]^ However, this only included 5 RCTs that applied the definition of POPF established by the ISGPF. In the ISGPF system, Grade B/C POPF is defined as clinically relevant POPF and requires more positive clinical intervention. In the ISGPF criteria, Grade A POPF has no impact on the clinical process. The meta-analysis of 5 RCTs showed no significant difference between duct-to-mucosa and invagination PJ in terms of CR-POPF. However, previous studies have provided conflicting results for this outcome. A meta-analysis involving 5 RCTs showed that invagination PJ appears to reduce CR-POPF rates.^[42]^ However, the original study included in this study did not adopt a unified definition of POPF. Similar to our study, the study conducted by Kilambi and Singh^[40]^ showed that duct-to-mucosa PJ does not appear to be better than invagination PJ. Our study's strength lies in incorporating the latest and most comprehensive RCTs. The study reported by Han et al^[44]^ was excluded because it was published in Chinese and did not provide enough data. A recent trial published by Bai et al^[15]^ demonstrated that duct-to-mucosa PJ seems to be better than invagination PJ. One of the factors that affects the development of POPF is the pancreatic texture. An RCT of patients with a soft pancreas conducted by Senda et al^[24]^ showed that invagination PJ was associated with lower rates of POPF and CR-POPF. Retrospective studies have shown that invagination PJ is more suitable for patients with soft pancreatic tissue and a smaller pancreatic duct diameter.^[45,46]^ According to a position statement by the ISGPF, no specific technique can reduce the incidence of POPF and CR-POPF.^[47]^ Future studies on this topic are therefore recommended.

DGE, which is usually not life-threatening, can increase patient discomfort, LOS, and medical costs. As with POPF, the definition of DGE varies among studies. POPF can increase the incidence of DGE. The DGE rates in the studies of the present meta-analysis ranged from 1.7% to 26.4%, and the rates between the 2 groups were similar. PPH is one consequence of CR-POPF; however, the definition of PPH varies among previous studies. The *I*^2^ test of the RCTs in the present analysis showed no heterogeneity (*I*^2^ = 0) among all studies and among studies using the ISGPF definition. Severe complications including severe POPF, bleeding, and abscess formation may require reoperation. The incidence of reoperation is an indicator for evaluating the safety of the PJ method. No significant difference in the reoperation rate was found in our meta-analysis. The overall rates of morbidity and mortality also vary among different studies. Similar to previous RCTs and meta-analyses,^[48,49]^ the morbidity and mortality rates were not significantly differences between the 2 groups.

The indications for PD in the included trials were heterogeneous. The most common indication was malignant disease. Few studies to date have focused on the long-term effects of tumors and the differences in residual pancreatic function between the 2 anastomotic methods. Studies have shown that catheter-to-mucosal anastomosis may cause catheter obstruction, leading to insufficient pancreatic function.

This meta-analysis had 2 main limitations. First, the details of the duct-to-mucosa and invagination techniques were variable among previous studies. Second, the usefulness of external stents and somatostatin and the patients’ clinical characteristics showed heterogeneity. Give these limitations, further RCTs on this topic are required.

## 5 Conclusion

Our study showed that duct-to-mucosa PJ is comparable with invagination PJ in terms of POPF, CR-POPF, and other main outcomes. Considering the above-mentioned limitations, high-quality RCTs are necessary in the future.

## Acknowledgment

The authors thank Angela Morben, DVM, ELS, from Liwen Bianji, Edanz Editing China (www.liwenbianji.cn/ac), for editing the English text of a draft of this manuscript.

## Author contributions

Conception and design of the study: Yunxiao Lyu. Studies selection: Ting Li and Yunxiao Lyu. Data extraction: Yunxiao Cheng and Bin Wang. Statistical analyses: Yunxiao Lyu and Sicong Zhao. Wrote the paper: Ting Li. The paper was revised and approved by Yunxiao Lyu.

**Conceptualization:** Yunxiao Lyu.

**Data curation:** Ting Li, Bin Wang, Yunxiao Cheng.

**Investigation:** Yunxiao Lyu.

**Software:** Yunxiao Lyu, Sicong Zhao.

**Writing – original draft:** Yunxiao Lyu, Ting Li.

**Writing – review & editing:** Yunxiao Lyu.

Yunxiao Lyu orcid: 0000-0002-2795-9698
